# Randomized controlled trial of postoperative exercise rehabilitation program after lumbar spine fusion: study protocol

**DOI:** 10.1186/1471-2474-13-123

**Published:** 2012-07-20

**Authors:** Sami Tarnanen, Marko H Neva, Joost Dekker, Keijo Häkkinen, Kimmo Vihtonen, Liisa Pekkanen, Arja Häkkinen

**Affiliations:** 1 Department of Health Sciences, University of Jyväskylä, Jyväskylä, Finland; 2 Department of Orthopaedic and Trauma Surgery, Tampere University Hospital, Tampere, Finland; 3 Department of Rehabilitation Medicine, VU University Medical Center, Amsterdam, The Netherlands; 4 Department of Biology of Physical activity, University of Jyväskylä, Jyväskylä, Finland; 5 Department of Orthopaedics and Traumatology, Central Finland Central Hospital, Jyväskylä, Finland; 6 Department of Physical Medicine and Rehabilitation, Central Finland Central Hospital, Jyväskylä, Finland

**Keywords:** Lumbar fusion, Disability, Pain, Quality of life, Spine, Exercise, Rehabilitation

## Abstract

**Background:**

Lumbar spine fusion (LSF) effectively decreases pain and disability in specific spinal disorders; however, the disability rate following surgery remains high. This, combined with the fact that in Western countries the number of LSF surgeries is increasing rapidly it is important to develop rehabilitation interventions that improve outcomes.

**Methods/design:**

In the present RCT-study we aim to assess the effectiveness of a combined back-specific and aerobic exercise intervention for patients after LSF surgery. One hundred patients will be randomly allocated to a 12-month exercise intervention arm or a usual care arm. The exercise intervention will start three months after surgery and consist of six individual guidance sessions with a physiotherapist and a home-based exercise program. The primary outcome measures are low back pain, lower extremity pain, disability and quality of life. Secondary outcomes are back function and kinesiophobia. Exercise adherence will also be evaluated. The outcome measurements will be assessed at baseline (3 months postoperatively), at the end of the exercise intervention period (15 months postoperatively), and after a 1-year follow-up.

**Discussion:**

The present RCT will evaluate the effectiveness of a long-term rehabilitation program after LSF. To our knowledge this will be the first study to evaluate a combination of strength training, control of the neutral lumbar spine position and aerobic training principles in rehabilitation after LSF.

**Trial registration:**

ClinicalTrials.gov Identifier NCT00834015

## Background

During the last 10 years there has been a significant increase in the number of lumbar spine fusions (LSF) [1]. The most common reasons for LSF are isthmic or degenerative spondylolisthesis, degenerative disc disease, and spinal stenosis [2]. In adult patients with lumbar isthmic or degenerative spondylolisthesis LSF has been reported to reduce symptoms [3,4]. However, the overall disability of patients after LSF may be high [5] and even 25% of patients rated the overall outcome as unchanged or worse in a 2-year follow-up study [3]. Most of the previous studies on LSF have evaluated the surgical procedure itself or compared conservative treatment to operative treatment. Less information is available on long-term exercise programs for patients after LSF surgery.

The effectiveness of rehabilitation after LSF has only been evaluated in four studies [6-9]. In these studies, the timing of the intervention has differed. In the studies of Nielsen et al. [8,9], prehabilitation started 6 to 8 weeks before surgery and continued during hospitalization. Abbott et al. [6] evaluated the effectiveness of psychomotor therapy implemented during the first 12 postoperative weeks. A Danish study [7] compared three different postoperative rehabilitation programs lasting between 12 and 20 postoperative weeks.

Exercise was an essential component of the rehabilitation protocols in all the LSF rehabilitation studies; however the guidance and exercise methods used were different. In the studies of Nielsen et al. and Christensen et al. [7-9], exercise programs included muscle endurance and strength training for the back and abdominal muscles, and cardiovascular conditioning. In the study of Abbott et al. [6], the exercise program consisted of motor relearning training of the transversus abdominis and multifidus, with cognitive and behavioral elements also integrated into the program. The results of these studies indicate that exercise may improve the outcome of LSF.

Typically, patients with lumbar isthmic or degenerative spondylolisthesis undergoing LSF have suffered low back pain for years and therefore may exhibit changes in the function [10] and structure of their trunk muscles [11], and in their cardiorespiratory condition [12]. LSF itself causes changes in the biomechanics of the lumbar spine, which may also accelerate degenerative changes in the adjacent segments [13] and cause muscle atrophy, leading to fatty infiltration of the lumbar muscles, especially in the multifidus[14-16]. As a possible consequence of these changes, low trunk muscle strength levels in patients after lumbar fusion have been reported [17,18].

The primary goals of the post-operative rehabilitation program are to control pain, decrease disability, restore back function, improve health related fitness and learn to use the low back during the healing process. Although the existing evidence supports the use of exercise in the rehabilitation of LSF patients, there is no consensus on the content of an exercise rehabilitation program after LSF. In addition, the durations of earlier interventions have been too short to achieve long-term changes in back function. Thus, there is a need to develop and test multifaceted rehabilitation programs to improve both back-specific and overall outcome after LSF. In contrast with previous exercise interventions for LSF patients, this study is novel in its development of a fusion-specific training program that takes into account changes in the biomechanics of the spine.

The main study questions are:

· Is combined back-specific and aerobic training more effective in decreasing back pain and disability than conventional instructions in postoperative rehabilitation?

· What are the effects of surgery and training on trunk muscle strength and mobility of the spine?

· What is the effect of fear of movement on post-operative exercise adherence, physical activity, pain and disability?

## Methods/design

### Study design

**Figure 1:**
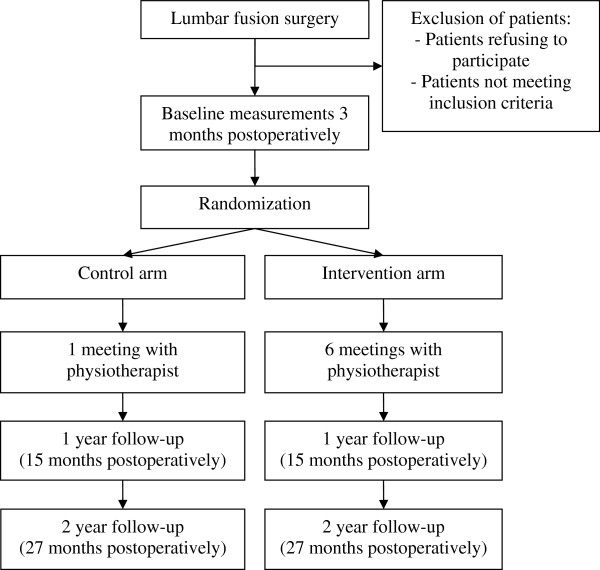
Flowchart of the study.

Figure 1 presents a flowchart of the study. The present randomized controlled trial will be conducted in Tampere University Hospital and the Central Finland Central Hospital. Approval of the study protocol was given by the Ethics Committee of the Central Finland Health Care District in 2008 (Dnro 4E/2008) and by the Ethics Committee of Tampere University Hospital in August 2008. Written informed consent will be obtained from all patients prior to participation.

### Participants

#### Inclusion criteria

All patients aged over 18 years scheduled to undergo elective LSF surgery for isthmic or degenerative spondylolisthesis in Tampere University Hospital or the Central Finland Central Hospital are eligible for the study. Patients will be recruited by the spine surgeons in each hospital.

#### Exclusion criteria

Patients with severe cardiorespiratory or musculoskeletal disease, severe psychiatric/psychological disorder, extensive lower limb paresis, social reasons (alcohol abuse), and immediate complications after back surgery (infection) will be excluded from the study.

### Surgery procedures

Spine surgeons will make the decision to operate according to their normal practice. The surgical procedure to be used is decompression and instrumented posterolateral fusion (PLF) with or without posterior lumbar interbody fusion (PLIF).

### Randomization and blinding

After surgery, the participants will be randomized into either the combined back-specific (combination of strength training and training of control of the neutral lumbar spine position) and aerobic training arm or to the control arm. The allocation will be based on computer randomization in blocks of four patients. The randomization will be performed and the randomization lists maintained by the research nurses, who will not be involved in the assessment or treatment of the participants. The first list will be used to randomize the participants with isthmic spondylolisthesis and the second list to randomize those with degenerative spondylolisthesis. Both centres will have their own randomization lists. Assessors will be blind to the treatment group in both study centres. Physiotherapists will not be blind to group membership; instead, but both study arms will have their own physiotherapist who will carry out postoperative guidance. Blinding the patients to the allocation is not possible due to the nature of the intervention.

### Preadmission clinic and early postoperative rehabilitation before the intervention

At the preadmission clinic, patients will meet with the spine surgeon, anesthesiologist, and physiotherapist, and be informed about the operation and rehabilitation. The early postoperative mobilization of the patients in the orthopaedic ward will be carried out by the physiotherapist. During the first three post-operative months, patients will be encouraged to walk and perform light abdominal, back, and thigh muscle exercises; stretching of hip muscles will also be included in the exercise program. The early postoperative exercise instructions will be similar for both study arms. The use of a bicycle ergometer will be allowed one month after the operation. Other types of exercise such as skiing, dancing, and water gymnastics will be permitted two months after surgery.

### Study arms

The intervention arms will start three months postoperatively and will last 12 months.

### Development of the intervention arm program

In the development of the protocol for the intervention arm, we have used information obtained from our own trunk muscle electromyography studies, conducted among healthy subjects [19,20] and lumbar fusion patients (Tarnanen et al., unpublished observation), other previously published studies on trunk and hip muscle activation during exercises [21-24], as well as information from a multidisciplinary group in the study hospitals (physiotherapists, nurses, spine surgeons), and feedback from patients regarding the feasibility of the program. The timing of the beginning of intervention is based on recovery from the surgery.

The back-specific exercise program has two main aims: (i) to improve control of the neutral lumbar spine position and (ii) increase trunk and hip muscle coordination, strength, and endurance [25-29]. (Table 1).

**Table 1:** Back-specific exercises program

**Phase**	**Back specific exercises**	**Goal of the exercise**
I	1. Squat (SP, EB)	MS
	2. Abdominal crunch (SUP)	ME
	3. Hip abduction (CLP)	CNSP
	4. Hip abduction and external rotation (SLP, EB)	CNSP/ME
	5. Hip extension (PRO)	CNSP
	6. Hip extension (FPKP, EB) Sets x Repetitions: 2 x 10-15-20	CNSP/ME
II	1. Squat (SP, EB)	MS
	2. &3. Bilateral shoulder extension and flexion (SP, EB)	ME/MS
	4. Heel slide or leg lift and knee extension with one leg (SUP)	CNSP
	5. Hip extension or hip extension and knee extension (CLP)	CNSP/ME
	6. Hip abduction (SLP, EB)	CNSP/ME
	7. Hip extension (EB) or bird dog exercise (FPKP) Sets x Repetitions: 2 x 10-15-20	CNSP/ME
III	1. Squat (SP, EB)	MS
	2. & 3. Bilateral shoulder extension and flexion (SP, EB)	ME/MS
	4. Leg lift and knee extension with one leg (SUP)	CNSP
	5. Hip extension and knee extension (CLP)	CNSP/ME
	6. Bird dog exercise (FPKP)	CNSP/ME
	7. Hip abduction (SP) Sets x Repetitions: 2–3 x 10-15-20	CNSP/ME
IV	1. Squat (EB) or forward lunge (SP)	MS
	2. Waiters bow exercise with elastic band (SP, EB)	MS
	3. & 4. Bilateral shoulder extension and flexion (SP, EB)	ME/MS
	5. & 6. Unilateral shoulder horizontal adduction and abduction (SIP, EB)	CNSP/ME
	7. Hip abduction (SP, EB) Sets x Repetitions: 2–3 x 10-15-20	CNSP/ME
V	1. Forward lunge (SP)	ME/MS
	2. Waiters bow exercise (SP, EB)	MS
	3. & 4. Unilateral shoulder horizontal adduction and abduction (SP, EB)	CNSP/ME
	5.& 6 Downward chop and upward chop (SIP, EB)	CNSP/ME
	7. Hip abduction (SP, EB) Sets x Repetitions: 2-3-4 x 10-15-20	CNSP/ME
VI	1. Forward lunge (SP)	ME/MS
	2. Waiters bow exercise (SP, EB)	MS
	3. & 4. Unilateral shoulder horizontal adduction and abduction (SP, EB)	CNSP/ME
	5. & 6. Downward chop and upward chop (SP, EB) Sets x Repetitions: 2-3-4 x 10-15-20	CNSP/ME

SP, standing position; SUP, supine position; CLP, crook lying position; SLP, side lying position; PRO, prone position; FPKP, four-point kneeling position; SIP, sitting position; EB, with elastic band resistance; MS, muscle strength; ME, muscle endurance; CNSP, control of the neutral lumbar spine position.

At the beginning of the program, trunk and hip muscle coordination and muscle endurance exercises will be performed in a prone, supine and four-point kneeling position. During the intervention the performance positions will gradually become more functional [30] and the loads increase progressively up to 50-70% of the repetition maximum to optimize muscle strength and muscle mass development. A subset of these exercises will be carried out with light loads to improve explosive force (high-velocity repetitions) and movement control. In addition, muscle-fatiguing training will be used for the back muscles to produce regional increases in blood flow capacity among the muscle fibers that experience increased activity during loading. Participants will be instructed to perform home exercises at least 2–3 times per week.

The aerobic walking program has three aims: (i) to increase the total amount of physical activity [31], (ii) improve patients’ aerobic capacity, and (iii) increase muscle capacity for fatty acid oxidation [32,33]. The program includes a progressive increase in the number of steps and interval walking workouts.

The total activity level will be evaluated during the first week by pedometers. Based on this information, patients will be instructed to increase their activity level progressively and monitor the amount of daily steps with the pedometer. (Table 2). Interval walking will be added to the exercise program four months after the beginning of the intervention. Each interval exercise consists of 5–10 minutes warm-up at normal walking speed, followed by periods of 30s - 1 min of brisk walking and 3 min of walking at normal speed alternated four times. The total length of the exercise bout will be 25–30 minutes. The length and intensity of brisk walking will be gradually increased during the last eight months.

**Table 2:** Aims for increasing the number of daily steps

**Aim**	**Model of progression**
10 000 steps/day, if: age under 65 years, healthy and no restrictions to increase physical activity	1. If baseline level <5 000 (sedentary), number of steps is increased 15% every other months until the target level is reached
	2. If baseline level 5 000–7 499 (”low active”), number of steps is increased 10% every other months until the target level is reached
	3. If baseline level 7 500–9 999 (”somewhat active”), number of steps is increased 5% every other months until the target level is reached
	4. If baseline level >10 000 (active), this level is maintained or number of steps is increased 5% every other months until 12 500/day (”highly active”) is reached (Categorized according to Tudor-Locke et al. 2008 [34])
7 500 steps/day, if: age >65 years and/or chronic diseases and/or some restriction to increase physical activity [35,36]	1. If baseline level <4 250, number of steps is increased 15% every other months until the target level is reached. In later phase this level is maintained or a new goal is set.
	2. If baseline level >4 250, number of steps is increased 10% every other months until the target level is reached. In later phase this level is maintained or a new goal is set.

Individual guidance sessions with the physiotherapist will be started three months after the LSF, with booster sessions every second month thereafter. In each session the physiotherapist will give guidance on the exercises to be performed in the next training phase and check the patients’ exercise techniques. In addition, patients will be given a leaflet containing written and pictorial information about the exercises. Each patient will perform the training independently at home; however, the progression of the exercises will be checked with the physiotherapist. During the first session, patients will fill in a personal exercise contract form and set their personal goals [37]. Goals will be reassessed in the middle phase of the intervention. Possible barriers to exercise (e.g. kinesiophobia) will be identified [38,39]. If a patient’s score on the Tampa scale for kinesiophobia (TSK) is over 37 in the post-operative assessment, the physiotherapist will explain to the patient (during the second/third guidance session) how and why some individuals with low back pain may develop a chronic pain syndrome (the fear-avoidance model, [40]). The patient’s experiences of the previous training phase will be reviewed during each guidance sessions. Patients will receive elastic bands (Thera-Band, The Hygenic Corporation, Akron Ohio, USA) and a pedometer (Omron Walking Style II, Kyoto, Japan) for their personal use.

### Control arm

Patients randomized to the control arm will be managed according to normal hospital rehabilitation practice. Three months postoperatively patients will receive instructions for home exercises in a single individual guidance session. The exercise program will consist of light muscle endurance (abdominal crunch, bird dog exercise, forward lunge, posterior pelvic tilt), mobility (hamstring stretch, lateral flexion of thoracic spine), and balance exercises (one-leg standing). Patients will be instructed to perform the home exercises 3 times per week.

### Outcomes

The outcome measurements will be assessed at baseline (3 months postoperatively), at the end of the exercise intervention period (15 months postoperatively), and after a 1-year follow-up. Only primary outcome variables will be used in the 27 months follow-up assessment.

#### Primary outcome variables

The intensity of back and lower limb pain during rest and daily activities in the past week will be assessed by means of the visual analogue scale (VAS) [41]. Disability due to back pain during the past week will be assessed by the Finnish version of the Oswestry Low Back Pain Disability Questionnaire 2.0 [42]. Quality of life will be evaluated by the Finnish version of the generic SF-36 Health Survey Questionnaire [43].

#### Secondary outcome variables

##### Physical function/fitness

Maximal isometric forces of the trunk flexors and extensors will be measured using a strain-gauge dynamometer [44]. Endurance strength of the trunk extensors will be measured by the Biering-Sorensen test [45,46]. Spinal mobility towards flexion will be measured by the Schober and Stibor tests [47] and fingertip–floor distance tests [45], and lateral bending by the method described by Frost et al. [48]. The intensity of pain during the trunk muscle strength and mobility measurements will be assessed with a VAS. The ‘timed up and go’ test (TUG) will be used to assess functional mobility (power, walk velocity, agility and dynamic balance) [49].

##### Kinesiophobia

The TSK will be used to measure the subjective experience of fear of movement [50].

##### Assessment of physical activity and exercise adherence

The amount of physical activity will be evaluated by the short form of the International Physical Activity Questionnaire (IPAQ) [51]. Training diaries will capture the frequency of the back-specific exercises and pedometers will be used to assess the total amount of daily steps in the intervention arm. The number of aerobic steps (10 minutes of continuous walking more than 60 steps per minute) during one week will be reported at least every second month.

## Statistical analysis

### Sample size

Cristensen et al. [7] estimated that a sample of ~60 patients (30 per group) is necessary to achieve 85% power for detecting a 25% difference in disability over time (baseline to 1 year), or at a follow-up of a 1 year, with a one-sided significance α-level of 0.05. However, we assume the between-group difference in pain will be lower in our participants. Assuming a dropout rate of 15-20% at the 1-year follow-up, we aim to include at least 80 patients (preferably 100) in our sample.

The clinical outcome variables will be analyzed by the intention-to-treat principle with the last observation carried forward (LOCF). The normality of variables will be evaluated by the Shapiro-Wilk statistic. Statistical comparison between the arms will be done using the chi-square test, Fisher's exact test, bootstrap-type analysis of covariance (ANCOVA) or multivariate analysis of variance (MANOVA) with Pillai’s trace statistics. A multiple imputation (Markov-chain Monte Carlo) method will be applied to supply possible missing values of individual questionnaire items, when appropriate.

## Discussion

This paper describes the rationale and design of a study which will assess the effectiveness of long-term combined back-specific (combination of strength training and training of control of the neutral lumbar spine position) and aerobic training in post-operative rehabilitation after lumbar spine fusion. Previous studies evaluating rehabilitation after LSF surgery are short-term and mostly focus on a specific type of exercise. However, trunk muscle function and health related fitness in patients with chronic low back pain are often so extensively impaired that more comprehensive training is probably needed. The effectiveness of exercise interventions are partly adherence-dependent, and thus special attention will be paid to patients goal setting, monitoring of progression and motivation. The selection of patients aims to reflect the patient population which usually undergoes this operation, and hence we will not be applying any strict exclusion criteria concerning age or comorbidities. This will improve the generalizability and implementability of the results. The results will have practical value in the planning and development of treatment options after lumbar spine fusion.

## Abbreviations

LSF, Lumbar spine fusion; VAS, Visual analogue scale; TSK, Tampa scale for kinesiophobia.

## Competing interests

The authors declare that they have no competing interests.

## Authors' contributions

ST, MHN, JD, KH, KV, LP and AH were responsible for the design of the study. All authors were involved in drafting the manuscript and revising it for critically important content. All authors have read and approved the final manuscript.

## Pre-publication history

The pre-publication history for this paper can be accessed here:

http://www.biomedcentral.com/1471-2474/13/123/prepub

## References

[B1] Rajaee SS Bae HW Kanim LE Delamarter RB Spinal fusion in the United States: analysis of trends from 1998 to 2008 Spine (Phila Pa 1976) 2012 37 1 67 76 10.1097/BRS.0b013e31820cccfb 21311399

[B2] Deyo RA Gray DT Kreuter W Mirza S Martin BI United States trends in lumbar fusion surgery for degenerative conditions Spine (Phila Pa 1976) 2005 30 12 1441 5 10.1097/01.brs.0000166503.37969.8a 15959375

[B3] Moller H Hedlund R Surgery versus conservative management in adult isthmic spondylolisthesis--a prospective randomized study: part 1 Spine (Phila Pa 1976) 2000 25 13 1711 1715 10.1097/00007632-200007010-00016 10870148

[B4] Weinstein JN Lurie JD Tosteson TD Hanscom B Tosteson AN Blood EA Birkmeyer NJ Hilibrand AS Herkowitz H Cammisa FP Albert TJ Emery SE Lenke LG Abdu WA Longley M Errico TJ Hu SS Surgical versus nonsurgical treatment for lumbar degenerative spondylolisthesis N Engl J Med 2007 356 22 2257 2270 10.1056/NEJMoa070302 17538085 PMC2553804

[B5] Maghout Juratli S Franklin GM Mirza SK Wickizer TM Fulton-Kehoe D Lumbar fusion outcomes in Washington State workers' compensation Spine (Phila Pa 1976) 2006 31 23 2715 2723 10.1097/01.brs.0000244589.13674.11 17077741

[B6] Abbott A Tyni-Lenne R Hedlund R Early rehabilitation targeting cognition, behavior, and motor function after lumbar fusion: a randomized controlled trial Spine (Phila Pa 1976) 2010 35 8 848 857 10.1097/BRS.0b013e3181d1049f 20354468

[B7] Christensen FB Laurberg I Bunger CE Importance of the back-cafe concept to rehabilitation after lumbar spinal fusion: a randomized clinical study with a 2-year follow-up Spine (Phila Pa 1976) 2003 28 23 2561 2569 10.1097/01.BRS.0000097890.96524.A1 14652472

[B8] Nielsen PR Andreasen J Asmussen M Tonnesen H Costs and quality of life for prehabilitation and early rehabilitation after surgery of the lumbar spine BMC Health Serv Res 2008 8 209 10.1186/1472-6963-8-209 18842157 PMC2586633

[B9] Nielsen PR Jorgensen LD Dahl B Pedersen T Tonnesen H Prehabilitation and early rehabilitation after spinal surgery: randomized clinical trial Clin Rehabil 2010 24 2 137 148 10.1177/0269215509347432 20103575

[B10] van Dieen JH Selen LP Cholewicki J Trunk muscle activation in low-back pain patients, an analysis of the literature J Electromyogr Kinesiol 2003 13 4 333 351 10.1016/S1050-6411(03)00041-5 12832164

[B11] Danneels LA Vanderstraeten GG Cambier DC Witvrouw EE De Cuyper HJ CT imaging of trunk muscles in chronic low back pain patients and healthy control subjects Eur Spine J 2000 9 4 266 272 10.1007/s005860000190 11261613 PMC3611341

[B12] Smeets RJ Wittink H Hidding A Knottnerus JA Do patients with chronic low back pain have a lower level of aerobic fitness than healthy controls?: are pain, disability, fear of injury, working status, or level of leisure time activity associated with the difference in aerobic fitness level? Spine (Phila Pa 1976) 2006 31 1 90 7 discussion 98 10.1097/01.brs.0000192641.22003.83 16395183

[B13] Ekman P Moller H Shalabi A Yu YX Hedlund R A prospective randomised study on the long-term effect of lumbar fusion on adjacent disc degeneration Eur Spine J 2009 18 8 1175 1186 10.1007/s00586-009-0947-3 19337757 PMC2899511

[B14] Fan S Hu Z Zhao F Zhao X Huang Y Fang X Multifidus muscle changes and clinical effects of one-level posterior lumbar interbody fusion: minimally invasive procedure versus conventional open approach Eur Spine J 2010 19 2 316 324 10.1007/s00586-009-1191-6 19876659 PMC2899808

[B15] Hyun SJ Kim YB Kim YS Park SW Nam TK Hong HJ Kwon JT Postoperative changes in paraspinal muscle volume: comparison between paramedian interfascial and midline approaches for lumbar fusion J Korean Med Sci 2007 22 4 646 651 10.3346/jkms.2007.22.4.646 17728503 PMC2693813

[B16] Motosuneya T Asazuma T Tsuji T Watanabe H Nakayama Y Nemoto K Postoperative change of the cross-sectional area of back musculature after 5 surgical procedures as assessed by magnetic resonance imaging J Spinal Disord Tech 2006 19 5 318 322 10.1097/01.bsd.0000211205.15997.06 16826001

[B17] Keller A Brox JI Gunderson R Holm I Friis A Reikeras O Trunk muscle strength, cross-sectional area, and density in patients with chronic low back pain randomized to lumbar fusion or cognitive intervention and exercises Spine (Phila Pa 1976) 2004 29 1 3 8 10.1097/01.BRS.0000103946.26548.EB 14699268

[B18] Tiusanen H Hurri H Seitsalo S Osterman K Harju R Functional and clinical results after anterior interbody lumbar fusion Eur Spine J 1996 5 5 288 292 10.1007/BF00304342 8915632

[B19] Tarnanen SP Ylinen JJ Siekkinen KM Malkia EA Kautiainen HJ Hakkinen AH Effect of isometric upper-extremity exercises on the activation of core stabilizing muscles Arch Phys Med Rehabil 2008 89 3 513 521 10.1016/j.apmr.2007.08.160 18295631

[B20] Tarnanen SP Siekkinen KM Hakkinen AH Malkia EA Kautiainen HJ Ylinen JJ Core Muscle Activation during Dynamic Upper Limb Exercises in Women J Strength Cond Res 2012 In press 10.1519/JSC.0b013e318248ad54 22222323

[B21] Distefano LJ Blackburn JT Marshall SW Padua DA Gluteal muscle activation during common therapeutic exercises J Orthop Sports Phys Ther 2009 39 7 532 540 19574661 10.2519/jospt.2009.2796

[B22] Ekstrom RA Donatelli RA Carp KC Electromyographic analysis of core trunk, hip, and thigh muscles during 9 rehabilitation exercises J Orthop Sports Phys Ther 2007 37 12 754 762 18560185 10.2519/jospt.2007.2471

[B23] Arokoski JP Valta T Airaksinen O Kankaanpaa M Back and abdominal muscle function during stabilization exercises Arch Phys Med Rehabil 2001 82 8 1089 1098 10.1053/apmr.2001.23819 11494189

[B24] Davidson KL Hubley-Kozey CL Trunk muscle responses to demands of an exercise progression to improve dynamic spinal stability Arch Phys Med Rehabil 2005 86 2 216 223 10.1016/j.apmr.2004.04.029 15706546

[B25] Akuthota V Nadler SF Core strengthening Arch Phys Med Rehabil 2004 85 3 Suppl 1 S86 92 15034861 10.1053/j.apmr.2003.12.005

[B26] McGill S Low back disorders: evidence-based prevention and rehabilitation 2007 2 Human Kinetics, Champaign, IL cop

[B27] Reiman MP Trunk stabilization training: an evidence basis for the current state of affairs J Back Musculoskelet Rehabil 2009 22 3 131 142 20023342 10.3233/BMR-2009-0226

[B28] Reiman MP Weisbach PC Glynn PE The hips influence on low back pain: a distal link to a proximal problem J Sport Rehabil 2009 18 1 24 32 19321904 10.1123/jsr.18.1.24

[B29] Suni J Rinne M Natri A Statistisian MP Parkkari J Alaranta H Control of the lumbar neutral zone decreases low back pain and improves self-evaluated work ability: a 12-month randomized controlled study Spine (Phila Pa 1976) 2006 31 18 E611 20 10.1097/01.brs.0000231701.76452.05 16915076

[B30] Norris C Matthews M The role of an integrated back stability program in patients with chronic low back pain Complement Ther Clin Pract 2008 14 4 255 263 10.1016/j.ctcp.2008.06.001 18940712

[B31] Hillsdon M Foster C Thorogood M Interventions for promoting physical activity Cochrane Database Syst Rev 2005 1 1 CD003180 15674903 10.1002/14651858.CD003180.pub2 PMC4164373

[B32] Talanian JL Galloway SD Heigenhauser GJ Bonen A Spriet LL Two weeks of high-intensity aerobic interval training increases the capacity for fat oxidation during exercise in women J Appl Physiol 2007 102 4 1439 1447 17170203 10.1152/japplphysiol.01098.2006

[B33] Nemoto K Gen-no H Masuki S Okazaki K Nose H Effects of high-intensity interval walking training on physical fitness and blood pressure in middle-aged and older people Mayo Clin Proc 2007 82 7 803 811 10.4065/82.7.803 17605959

[B34] Tudor-Locke C Hatano Y Pangrazi RP Kang M Revisiting "how many steps are enough?" Med Sci Sports Exerc 2008 40 7 Suppl S537 43 18562971 10.1249/MSS.0b013e31817c7133

[B35] Tudor-Locke C Bassett DR How many steps/day are enough? Preliminary pedometer indices for public health Sports Med 2004 34 1 1 8 10.2165/00007256-200434010-00001 14715035

[B36] Tudor-Locke C Washington TL Hart TL Expected values for steps/day in special populations Prev Med 2009 49 1 3 11 10.1016/j.ypmed.2009.04.012 19409409

[B37] Åsenlöf P Denison E Lindberg P Behavioral goal assessment in patients with persistent musculoskeletal pain Physiother Theory Pract 2004 20 4 243 254 10.1080/09593980490887957

[B38] Rhodes RE Fiala B Building motivation and sustainability into the prescription and recommendations for physical activity and exercise therapy: the evidence Physiother Theory Pract 2009 25 5–6 424 441 19842866 10.1080/09593980902835344

[B39] Jordan JL Holden MA Mason EJE Foster NE Interventions to improve adherence to exercise for chronic musculoskeletal pain in adults Cochrane Database Syst Rev 2010 1 1 CD005956 20091582 10.1002/14651858.CD005956.pub2 PMC6769154

[B40] Leeuw M Goossens ME Linton SJ Crombez G Boersma K Vlaeyen JW The fear-avoidance model of musculoskeletal pain: current state of scientific evidence J Behav Med 2007 30 1 77 94 10.1007/s10865-006-9085-0 17180640

[B41] Dixon JS Bird HA Reproducibility along a 10 cm vertical visual analogue scale Ann Rheum Dis 1981 40 1 87 89 10.1136/ard.40.1.87 7469530 PMC1000664

[B42] Pekkanen L Kautiainen H Ylinen J Salo P Hakkinen A Reliability and validity study of the Finnish version 2.0 of the oswestry disability index Spine (Phila Pa 1976) 2011 36 4 332 338 20823785 10.1097/BRS.0b013e3181cdd702

[B43] Aalto A Rand 36-item health survey 1,0: suomenkielinen versio terveyteen liittyvän elämänlaadun kyselystä: kyselylomake ja käyttöohjeet 1995 Stakes, Helsinki

[B44] Rantanen P Airaksinen O Penttinen E Paradoxical variation of strength determinants with different rotation axes in trunk flexion and extension strength tests Eur J Appl Physiol Occup Physiol 1994 68 4 322 326 10.1007/BF00571451 8055890

[B45] Biering-Sorensen F Physical measurements as risk indicators for low-back trouble over a one-year period Spine (Phila Pa 1976) 1984 9 2 106 119 10.1097/00007632-198403000-00002 6233709

[B46] Latimer J Maher CG Refshauge K Colaco I The reliability and validity of the Biering-Sorensen test in asymptomatic subjects and subjects reporting current or previous nonspecific low back pain Spine (Phila Pa 1976) 1999 24 20 2085 9 discussion 2090 10.1097/00007632-199910150-00004 10543003

[B47] Macrae IF Wright V Measurement of back movement Ann Rheum Dis 1969 28 6 584 589 10.1136/ard.28.6.584 5363241 PMC1031291

[B48] Frost M Stuckey S Smalley LA Dorman G Reliability of measuring trunk motions in centimeters Phys Ther 1982 62 10 1431 1437 7122701 10.1093/ptj/62.10.1431

[B49] Podsiadlo D Richardson S The timed "Up & Go": a test of basic functional mobility for frail elderly persons J Am Geriatr Soc 1991 39 2 142 148 1991946 10.1111/j.1532-5415.1991.tb01616.x

[B50] Vlaeyen JW Kole-Snijders AM Boeren RG van Eek H Fear of movement/(re)injury in chronic low back pain and its relation to behavioral performance Pain 1995 62 3 363 372 10.1016/0304-3959(94)00279-N 8657437

[B51] Craig CL Marshall AL Sjostrom M Bauman AE Booth ML Ainsworth BE Pratt M Ekelund U Yngve A Sallis JF Oja P International physical activity questionnaire: 12-country reliability and validity Med Sci Sports Exerc 2003 35 8 1381 1395 10.1249/01.MSS.0000078924.61453.FB 12900694

